# Comparative genomic assessment of Multi-Locus Sequence Typing: rapid accumulation of genomic heterogeneity among clonal isolates of *Campylobacter jejuni*

**DOI:** 10.1186/1471-2148-8-229

**Published:** 2008-08-08

**Authors:** Eduardo N Taboada, Joanne M MacKinnon, Christian C Luebbert, Victor PJ Gannon, John HE Nash, Kris Rahn

**Affiliations:** 1 Laboratory for Foodborne Zoonoses (Lethbridge Unit), Public Health Agency of Canada c/o Animal Diseases Research Institute, PO Box 640, Township Road 9-1, Lethbridge, Alberta, T1J 3Z4, Canada; 2 Laboratory for Foodborne Zoonoses, Public Health Agency of Canada, 110 Stone Road West, Guelph, Ontario, N1G 3W4, Canada; 3 Institute for Biological Sciences, National Research Council of Canada, 100 Sussex Drive, Ottawa, Ontario, K1A 0R6, Canada

## Abstract

**Background:**

Multi-Locus Sequence Typing (MLST) has emerged as a leading molecular typing method owing to its high ability to discriminate among bacterial isolates, the relative ease with which data acquisition and analysis can be standardized, and the high portability of the resulting sequence data. While MLST has been successfully applied to the study of the population structure for a number of different bacterial species, it has also provided compelling evidence for high rates of recombination in some species. We have analyzed a set of *Campylobacter jejuni *strains using MLST and Comparative Genomic Hybridization (CGH) on a full-genome microarray in order to determine whether recombination and high levels of genomic mosaicism adversely affect the inference of strain relationships based on the analysis of a restricted number of genetic loci.

**Results:**

Our results indicate that, in general, there is significant concordance between strain relationships established by MLST and those based on shared gene content as established by CGH. While MLST has significant predictive power with respect to overall genome similarity of isolates, we also found evidence for significant differences in genomic content among strains that would otherwise appear to be highly related based on their MLST profiles.

**Conclusion:**

The extensive genomic mosaicism between closely related strains has important implications in the context of establishing strain to strain relationships because it suggests that the exact gene content of strains, and by extension their phenotype, is less likely to be "predicted" based on a small number of typing loci. This in turn suggests that a greater emphasis should be placed on analyzing genes of clinical interest as we forge ahead with the next generation of molecular typing methods.

## Background

*Campylobacter jejuni *is the most common cause of acute bacterial enteritis worldwide [1,2]. Despite significant progress in recent years, critical gaps remain in our understanding of *C. jejuni *pathogenesis. The lack of a well-defined set of virulence determinants makes it difficult to assess the virulence potential of different strains or to make links between specific genotypes and specific disease manifestations. Similarly, because the majority of infections are sporadic, sources and routes of transmission remain unclear in most cases of campylobacteriosis [3].

Significant effort has been placed on the development of methods for the typing of *C. jejuni *based on the analysis of polymorphic DNA targets and that have been applied to the study of species diversity and in the context of epidemiology and surveillance [4,5]. The large number of competing approaches is a reflection on the fact that different methods may be appropriate for investigating short-term outbreak investigations (i.e. local epidemiology) and/or large-scale longitudinal surveillance (i.e. global epidemiology) [6]. Multi-locus sequence typing or MLST [7,8], which is based on the analysis of DNA sequence polymorphisms in a group of housekeeping genes, has recently emerged as a strong contender for a genotyping "gold standard" for *C. jejuni *on the strength of several features. These include: a high discriminative power; ease of standardization of data acquisition and analysis across laboratories, and the high portability of the resulting sequence data [4,9].

MLST benefits from a well-established framework for the phylogenetic analysis of molecular sequences. This has led to the suggestion that the phylogenetic signal contained within the loci analysed by MLST could be successfully used for long-term tracking in population structure studies, global epidemiology and long-term surveillance [10]. However, two outstanding questions need to be addressed in light of emerging data from comparative genomic analyses of *C. jejuni*. First, *C. jejuni *is naturally transformable and takes up homologous DNA readily [11], leading to high rates of intraspecies recombination [12,13] that could distort the genetic relationships inferred from any one genetic locus. Second, a potential weakness which MLST shares with most genotyping approaches is that strain relatedness is inferred based on a very limited sub-sampling of the entire genome [5,14]. This becomes increasingly relevant given the extensive genomic diversity that has been observed in intraspecies comparisons of *C. jejuni *through whole genome sequencing [15] and whole-genome microarray-based comparative genomic hybridization (CGH) [16-20].

MCGH has recently been successfully applied to the examination of gene conservation dynamics and to the investigation of strain to strain relationships based on whole-genome gene conservation profiles [21]. In this study, we have analyzed a set of strains using both MLST and MCGH in order to assess whether the strain relationships inferred from the seven loci interrogated by MLST are consistent with the phylogenetic signal obtained from the analysis of whole-genome comparative genomic data.

## Results

### Description of isolate relationships determined by MLST

**Figure 1:**
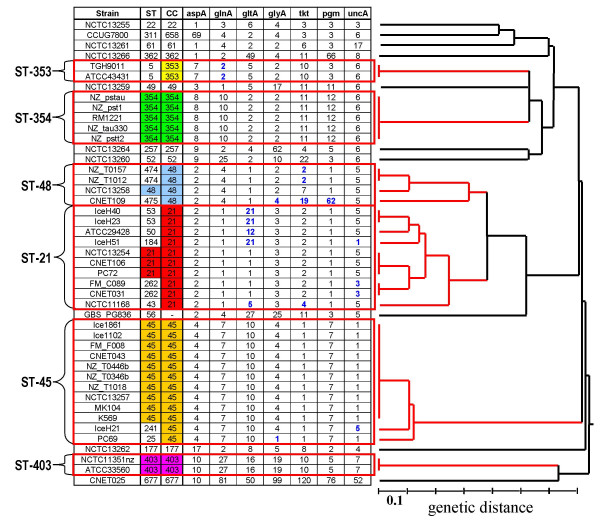
**UPGMA-based clustering of MLST data for the 45 *C. jejuni *strains included in this study**. Clusters representing clonal complexes (CC) are highlighted in red on the dendogram and their corresponding allelic profiles are also boxed in red. Allelic differences with respect to the central sequence type (ST) of the CC are highlighted in blue.

In order to evaluate relationships among isolates, all 45 strains in this study were analyzed by MLST (Figure 1). The strains were selected from a larger dataset analyzed by MLST and were picked to comprise several levels of genetic similarity, thus enabling us to determine whether relationships assessed by MLST would be supported by CHG data in the short-term vs. long-term epidemiological context.

The dataset contained representatives from 25 distinct Sequence Types (STs) with 8 STs containing multiple strains. BURST analysis identified two main lineages, clonal complexes ST-21 and ST-45, which figured prominently in the dataset. The strains from the ST-21 complex include strains of the ST-21, Single Locus Variants (SLVs) (ST-50, ST-53, and ST-262), and Double Locus Variants (DLVs) (ST-43 and ST-184). The ST-45 complex includes strains of the ST-45 and SLVs (ST-25 and ST-241).

A subset of strains with the ST-474, which was originally placed in the ST-21 complex based on preliminary BURST analysis on the strength of matches at 5 of the 7 typing loci, was subsequently re-assigned to the ST-48 complex based on lineage assignments obtained from the *C. jejuni *MLST database, which is based on a much larger dataset including data on over 3000 strains. The ST-48 complex includes strains with the ST-48, SLVs (ST-474) and a Triple Locus Variant (TLV) (ST-475). In addition to the strains from ST-21, ST-45, and ST-48 complexes, groups of strains with identical STs were found from three additional clonal complexes (ST-353, ST-354, and ST-403). The remaining 10 strains did not belong to any of the ST complexes represented in the dataset and shared at most 3 of 7 MLST alleles with their closest matches.

### Analysis of isolate relationships determined by MCGH

**Figure 2:**
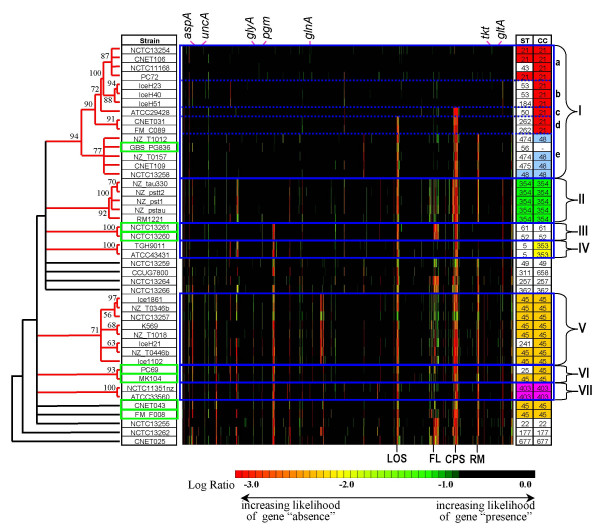
**Concordance between clustering of MLST and whole-genome CGH profiles for the 45 *C. jejuni *isolates included in this study**. Bootstrap support is shown for the statistically robust clusters (shown in red on the dendogram; CGH profiles boxed in blue). Log Ratio data has been colour-coded according to data interpretation thresholds described in Taboada *et al*. [31]. Strains showing discordant clustering results are boxed in green.

In order to assess strain-to-strain relationships based on genome similarity, gene conservation profiles derived from CGH data were used to quantify genomic similarity and this data was then used as a measure of the genetic distance between strains. Hierarchical clustering of the strains was performed on the resulting distance matrix of all pair wise distances between strains and bootstrap analysis revealed seven statistically robust clusters of strains (Figure 2) which were highly concordant with those obtained by hierarchical clustering of microarray profiles using the Pearson correlation metric (results not shown).

Cluster I can be further divided into 5 sub-types with significant differences in gene content: clusters Ia and Ib include the reference genome strain NCTC 11168 and six other strains with a very small number of genes displaying "significant Log Ratio differences" (SLRDs) with respect to NCTC 11168. These SLRDs correspond to likely gene divergence/gene absence events with respect to the reference strain. The three additional strains in cluster Ia have an average of 12.0 SLRDs whereas strains in cluster Ib have an average of 14.3 SLRDs. Strains in clusters Ic, Id, and Ie have an increasing number of differences with respect to the reference strain (an average of 37.3 SLRDs), although the majority of these are concentrated within four genetic loci: a region spanning Cj0968 to Cj0972 in cluster Ie; the lipo-oligosaccharide biosynthesis locus or LOS (Cj1136-Cj 1146c) in clusters Id and Ie; the capsular polysaccharide biosynthesis locus or CPS (Cj1414c-Cj1449) in clusters Ic, Id, and Ie; and a Type I restriction/modification locus or R-M (Cj1549-Cj1560) in cluster Ie.

Strains from clusters II to VII have an average number of SLRDs with respect to the reference strain that range from 64.5 for cluster IV to 97.9 for cluster V. The distribution and prevalence of various SLRDs across the genome varies substantially for each group, although the bulk can be found within the various hyper-variable loci previously described in *C. jejuni *[18,19]. For example: only strains from clusters I and II appear to have a fully conserved (i.e. lacking in SLRDs) region spanning Cj0480 to Cj0490; only strains from cluster V have SLRDs in the region spanning Cj0727 and Cj0741; only strains from clusters I, III and IV lack SLRDs within the Type I restriction/modification locus. It thus appears that the various clusters of strains are characterized by unique patterns of conserved genes at hyper-variable loci.

### Comparison of MCGH versus MLST-derived isolate relationships

When results of MCGH-based clustering were compared to the results obtained by MLST-based clustering and BURST analysis (Figure 2) we found that, with a few exceptions, the statistically robust groups obtained from CGH analysis correspond to groups of strains of identical sequence type. For example, strains in cluster Ia are largely of ST-21; strains from cluster 1b are largely of ST-53; strains from cluster 1d are of ST-262; strains from cluster II are of ST-354; strains from cluster IV are of ST-5; strains from cluster V are of ST-45; strains from cluster VII are of ST-403. In general, the congruence observed between CGH and MLST profiles also extends to strains within the same clonal complex (i.e. defined by sharing at least 4 loci) since strains with similar CGH profiles tend to share multiple MLST alleles. For example, strains in clusters Ia, Ib, Ic, and Id form part of clonal complex ST-21 and share 5 or more alleles. Similarly, the eight strains in cluster V share 6 or more alleles.

**Figure 3:**
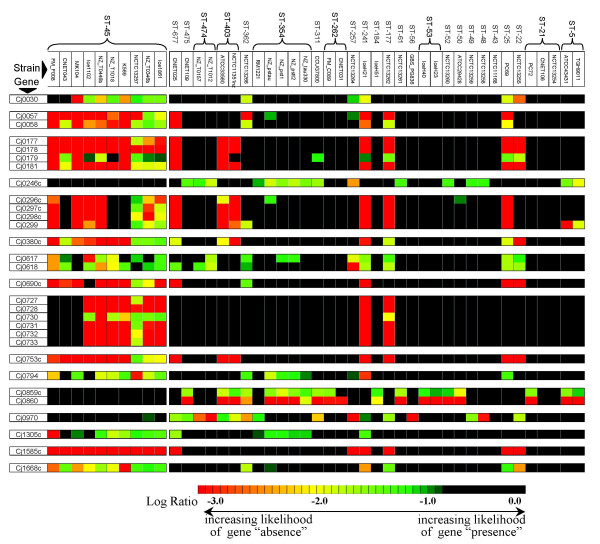
**Shared genomic attributes in strains from the same MLST clonal complex**. The strains of ST-45 show significant differences in gene conservation rates at the loci shown in the first column with respect to all other strains in the dataset and differentiate this group of genetically related strains from other groups of strains.

An inspection of local gene conservation patterns shows that strains from the same ST tend to share attributes that are nearly exclusive to the group (Figure 3). For example, whereas the ten strains with ST-45 have SLRDs at Cj0057 and Cj0058, this specific pattern is observed in only 5 of the remaining 35 strains in the dataset. Similarly, the pattern found among ST-45 strains in the region from Cj0177 to Cj0181 is found in only 7 of the remaining strains in the dataset. Other genomic regions where SLRDs are found almost exclusively among ST-45 strains are the multi-gene loci spanning Cj0296c to Cj0299, Cj0617-Cj0618, Cj0727 to Cj0733, and eight other single-gene loci (Cj0030, Cj0380c, Cj0690c, Cj0753c, Cj0794, Cj1305, Cj1585c, Cj1668c). A small number of genes (Cj0246c, Cj0859c, Cj0860, and Cj0970) appear to be fully conserved in all ST-45 strains but have SLRDs in a number of strains in the remainder of the dataset. More broadly, such differences in gene content can be used to differentiate the various CGH clusters and ST complexes.

Although our data suggests that strains with similar MLST profiles share similar CGH profiles, we have also found evidence for strains with significant levels of genomic similarity despite sharing few or none of the alleles used for MLST. For example, strain GBS_PG836 shows high overall genome similarity to the strains in the ST-48 complex (Cluster Ie) despite having different alleles at 4 of 7 MLST loci. In an extreme example, strains NCTC_13260 and NCTC_13261 show significant congruence in overall CGH profiles despite sharing no mutual alleles at any of the seven MLST loci. Thus, despite the fact that similarity in MLST profiles is generally a good predictor for genomic similarity, it is not always indicative of overall genome similarity between strains.

### Genomic heterogeneity within groups of strains with the same MLST sequence type

**Figure 4:**
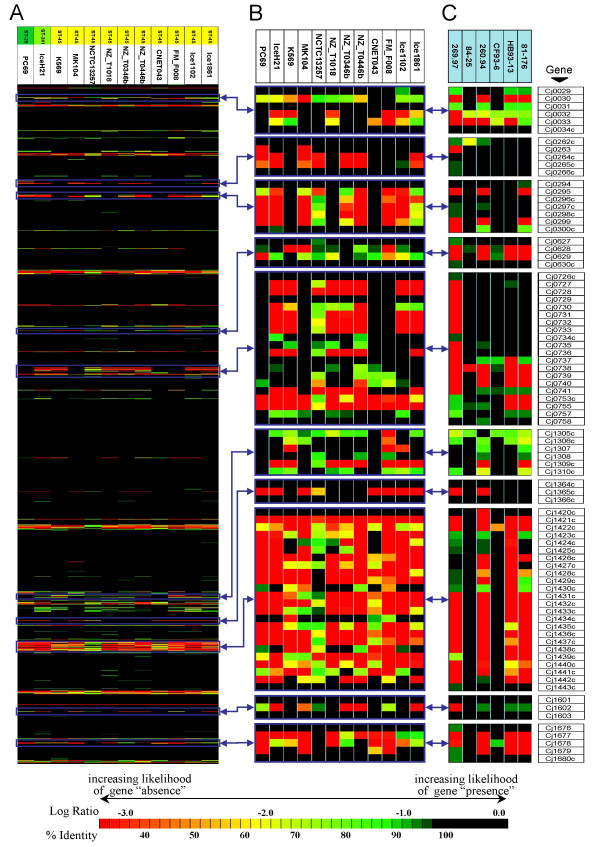
**An examination of genomic mosaicism within clonal complex ST-45**. Although strains within ST-45 have similar overall CGH profiles (A), significant genomic heterogeneity can be observed across various hyper-variable loci in the *C. jejuni *genome (B). Mosaicism observed in the CGH data is consistent with that observed in newly sequenced *C. jejuni *genomes (C). (note: Log Ratio data in (A) and (B) and sequence identity data in (C) were colour coded using a common scale reflecting the likelihood of gene presence/absence).

Although genetic relatedness is reflected by shared gene content and high similarity in overall CGH profiles, when global and local gene conservation profiles are examined it is also possible to observe significant differences in gene content between strains of the same CGH cluster/clonal complex, particularly within hyper-variable genomic loci. For example, an examination of the strains in the ST-45 complex reveals that each strain appears to contain a heterogeneous mixture of conserved and divergent/absent genes and thus a "mosaic" pattern of gene conservation is apparent even among strains with high overall genomic similarity that are members of the same MLST clonal complex (Figure 4a, 4b). Although, on average, strains from the ST-45 complex have more SLRDs with respect to other strains in the dataset (μ = 93 ± 13), they bear a significant number of SLRDs with respect to one another (μ = 65 ± 14). Similar observations can be made for the strains in the ST-354 complex (μ = 83 ± 15 vs. μ = 35 ± 15). [For a complete set of all pair-wise SLRDs consult Additional file 1]

To examine whether these apparent mosaic patterns of gene content do not merely represent an artifact of the MCGH technique, we examined and visualized gene conservation patterns of various hyper-variable loci among newly sequenced *C. jejuni *genomes (Figure 4c). This examination revealed that the hypervariable loci in these strains have highly heterogeneous gene content with conserved and absent/divergent genes interspersed, a pattern that is consistent with our microarray-CGH data.

### Disruption of genetic linkage in a genomic region flanked by two MLST loci

**Figure 5:**
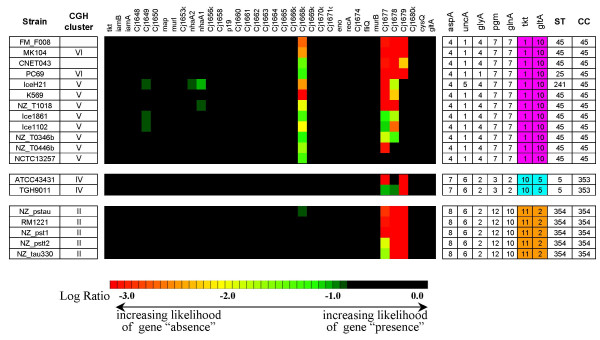
**An examination of genetic linkage in groups of genetically related strains**. Although strains with identical *tkt-gltA *alleles can also share similar gene conservation profiles within the intervening genomic region, disruption of linkage is apparent among members the same CC that share the same *tkt-gltA *loci.

We have exploited the relatively close proximity between two MLST loci in combination with microarray-derived comparative genomic data to examine genetic linkage in *C. jejuni*. The *tkt *(Cj1645) and *gltA *(Cj1682c) loci are located approximately 36 Kb apart in the *C. jejuni *NCTC 11168 genome and variability has been observed in several genes contained within the region flanked by these two genes [19]. We thus set out to examine whether we could find evidence for an association between specific allele combinations of *tkt *and *gltA *and gene conservation profiles in the intervening region among members of the same clonal complex. Although it is possible to observe similar gene conservation profiles for members of the same clonal complex sharing the same *tkt-gltA *allele combinations (data not shown), some genomic heterogeneity is also apparent (Figure 5).

## Discussion

Multi-locus Sequence Typing (MLST) has emerged as a leading molecular typing method for examining strain relationships which has been effectively applied to a number of different bacterial species [10], including *C. jejuni *[7,22]. Although MLST has been successfully applied to the study of the population structure of the *C. jejuni *it has also, paradoxically, provided compelling evidence that *C. jejuni *populations are subject to high rates of horizontal genetic exchange, with recombinational events contributing to a significant proportion of the allelic diversity observed [12,13].

The effects of high rates of intraspecies recombination observed include: a) conflicting phylogenetic signal obtained from different genes due to their different evolutionary trajectories, and b) a panmictic population structure for which clonal evolution is not the predominant trend. Both of these effects could pose limitations on the reliability of genetic relationships inferred from one or a small number of molecular markers as this small number of loci could themselves be subject to recombination. We have used microarray-based comparative genomic hybridization (MCGH) to analyze a collection of strains for which MLST analysis suggests varying levels of genetic relatedness. The dataset also includes clonal clusters comprised of strains with and without apparent epidemiological links in order to provide a "whole-genome" context for examining strain relationships inferred from MLST data.

An interesting finding from this study is the fact that global patterns of gene conservation obtained by MCGH are well correlated to MLST data. We have found that the robust clusters predicted by MCGH analysis (> 75% bootstrap support) and by MLST analysis (clonal complexes sharing 4 or more alleles) display excellent agreement (Figure 3). Of the 35 strains in our dataset that fall within six MLST clonal complexes only 4 fail to cluster robustly within a corresponding CGH cluster. Similarly, of the 37 strains that form statistically robust CGH clusters only 3 lack support from MLST data. Although the relationship between MLST genotypes and global-gene conservation profiles might be expected for strains with shared epidemiology, surprisingly this relationship appears to be evident for strains that do not share an obvious epidemiological connection. It is worth noting that the dendograms obtained for this dataset from the phylogenetic analysis of individual MLST loci are largely incongruent with one another (data not shown), likely due to recombination at the various alleles. Overall congruence between gene content and MLST has recently been observed for *Streptococcus pneumoniae*, another highly recombinogenic species [23]. In this light, the high degree of congruence between MLST and CGH data is not surprising and suggests that the multilocus approach appears to significantly mitigate the effects of lateral exchange in the examination of strain relationships.

It is important to stress that congruence between global gene conservation patterns and MLST genotypes does not preclude significant differences in gene content between related strains. Although, as expected, gene content differences tend to be highest between unrelated strains with greatest genetic distance (i.e. based on analysis of the MLST loci), our data provides evidence for significant genomic mosaicism between closely related strains through the accumulation of gene content differences. Thus, while related strains may share an increased number of genomic features, including a similar profile of significant SLRDs at any of a number of hyper-variable loci spread throughout the genome, their specific gene content in terms of absent and divergent genes may differ considerably. Although the widespread extent of this mosaic pattern of gene content might appear to be an artefact of the CGH technique we have employed in our analysis, evidence from comparative genomic sequencing would suggest otherwise. For example, mosaic patterns of gene conservation have been previously observed at a number of hyper-variable loci (LOS: [24,25]; CPS: [26]; and RM: [27]). Similarly, our preliminary examination of additional hyper-variable loci among newly sequenced *C. jejuni *genomes (Figure 5c) has yielded similar observations. An examination of the genomic region bracketed by the MLST loci *tkt *and *gltA *also demonstrates that genomic events altering local gene conservation profiles can occur among members of the same clonal complex that share the same alleles at these loci (Figure 5), implying that great care must be made in extrapolating the gene content of strains based on indirect observations made at a different set of genetic loci.

A common theme among comparative sequencing studies is the suggestion that recombination is a potent driving force shaping the gene conservation patterns of hyper-variable loci of *C. jejuni *through events such as allelic replacements, gene fusions, gene duplications and gene deletions. Analysis of MLST allele patterns further suggests that housekeeping loci are also targeted by recombination [12,13]. It thus appears that recombinational exchange in *C. jejuni *is not only widespread but that it must occur at significant frequencies consistent with the rapid accumulation of gene content differences we have observed among closely related strains in this study.

## Conclusion

Although our data suggest that reliable strain relationships can be inferred despite the rapid pace of genetic change due to recombination, our ability to couple molecular typing data to phenotypes of interest (e.g. virulence, drug resistance) may be restricted by the shifting gene content among related strains. An advantage of molecular characterization methods based on the comparison of gene content is illustrated by our recent analysis of *C. jejuni *strains implicated in Guillain-Barré and Miller Fisher syndromes [28]. Neuropathogenic *C. jejuni *have been highly refractory to analysis by conventional molecular typing because of their diverse lineage and due to the lack of association between conventional molecular typing markers and their clinical phenotype [29]. Although our whole-genome CGH analysis merely confirmed earlier observations regarding the population structure of neuropathogenic strains, it correctly identified a small number of genes whose presence among strains of diverse lineage is though to be highly correlated with a neuropathic clinical outcome [28].

The rapid assessment of neuropathogenic potential of strains has since been achieved by the directly targeting polymorphisms within the genes of interest [30]. Ultimately, the development of clinically relevant molecular typing approaches may be better served by comparative genomic methods that directly survey the genetic differences responsible for the phenotypes of interest rather than through indirect evidence from comparison of molecular typing targets unrelated to phenotype

## Methods

### Bacterial strains

Background on the 45 strains we analyzed by microarray CGH and MLST is presented in Table 1. The strains were selected from a larger dataset analyzed by MLST and picked to comprise several levels of genetic similarity, which would thus enable us to determine whether relationships assessed by MLST would be supported by CHG data in the short-term vs. long-term epidemiological context. Strains were picked to comprise several levels of genetic similarity, which would thus enable us to determine whether relationships assessed by MLST would be supported by CHG data.

**Table 1:** List of strains used for this study.

Strain	Serotype^3^	Source	MLST Sequence Type
ATCC29428	O:1	human	50
ATCC33560	O:23	cattle	403
ATCC43431	O:3	human	5
CCUG7800	O:4	human	311
CNET025	O:58	wild bird	677
CNET031	O:1	human	262
CNET043	O:58	human	45
CNET106	O:2	sheep	21
CNET109	O:4,50	canine	475
FM_C089	n.d.	human	262
FM_F008	n.d.	chicken	45
GBS_PG836	n.d.	human	56
Ice1102^1^	n.d.	chicken	45
Ice1861^1^	n.d.	chicken	45
IceH21^1^	n.d.	human	241
IceH23^1^	n.d.	human	53
IceH40^1^	n.d.	human	53
IceH51^1^	n.d.	human	184
K569	n.d.	chicken	45
MK104	O:19	human	45
NCTC11168	O:2	human	43
NCTC11351	O:23	cattle	403
NCTC13254	O:50	cattle	21
NCTC13255	O:19	human	22
NCTC13257	O:57	human	45
NCTC13258	O:50	ovine	48
NCTC13259	O:18	human	49
NCTC13260	O:5	ovine	52
NCTC13261	O:50	cattle	61
NCTC13262	NT	environment (sand)	177
NCTC13264	O:11	human	257
NCTC13266	O:41	human	362
NZ_pst1^2^	n.d.	chicken	354
NZ_pstau^2^	n.d.	chicken	354
NZ_pstt2^2^	n.d.	chicken	354
NZ_T1012^2^	n.d.	chicken	474
NZ_T1018^2^	n.d.	chicken	45
NZ_T157^2^	n.d.	chicken	474
NZ_T346b^2^	n.d.	chicken	45
NZ_T446b^2^	n.d.	chicken	45
NZ_tau330^2^	n.d.	chicken	354
PC69	O:9	human	25
PC72	O:2	human	21
RM1221	n.d.	chicken	354
TGH9011	O:3	human	5

^1 ^strains collected as part of "Campy-On-Ice" consortium

^2 ^strains collected as part of New Zealand study outlined in Pope *et al*. [43]

### DNA isolation

Cells were grown on Mueller-Hinton agar plates (BACTO, Oakville, ON) for 36 hours at 42°C under microaerophilic conditions prior to genomic DNA isolation. Genomic DNA was isolated by phenol:chloroform extraction as previously described [19]. For MLST, genomic DNA was prepared using the Qiagen Tissue Kit (Qiagen, Mississauga, ON) according to the manufacturer's instructions.

### Microarray Hybridizations

Details of the microarray, including primer selection, the parameters for primer synthesis, selection of amplicons, as well as the purification and printing of DNA onto slides were previously described elsewhere [19]. Hybridizations were performed using protocols described previously [31]. Briefly, for each tester strain equivalent amounts of Cy-3 labelled tester and Cy-5 labelled control genomic DNAs (i.e. strain NCTC 11168) with similar dye incorporation efficiencies were pooled and co-hybridized to our microarray.

### Microarray data acquisition and analysis

Microarrays were scanned using a Chipreader laser scanner (BioRad, Mississauga, ON) according to the manufacturer's recommendations. Spot quantification, visual inspection of potential outliers, and flagging of anomalous spots was performed using the program ArrayPro Analyzer (version 4.5; Media Cybernetics). The microarray data exported from ArrayPro was imported into the BioArray Software Environment (BASE version 1.2) [32] and is available at NCBI's Gene Expression Omnibus [33] under accession number GSE9919. Spots flagged due to poor spot morphology or low signal intensity (less than 3 X local background) were filtered out. After print-tip Loess normalization, data was used to calculate the average Log Ratio or LR (i.e. log_2 _[Signal Tester/Signal Control]) from the two replicates for each gene represented on the microarray. The filtered data exported from BASE contains the average LR data for 1606 genes.

### MCGH data analysis and visualization

LR data was visualized and analyzed in TIGR's MultiExperiment Viewer (MEV version 3.0) [34] with high-resolution heat maps of LR data generated using a custom-script written in VBA for MS-Excel; all CGH data was organized assuming synteny with *C. jejuni *NCTC 11168 in order to examine mapping of variable genes to genomic regions. Clustering of strains based on LR profile similarities was performed by the average linkage hierarchical clustering method [35], as implemented in TMEV, using Pearson correlation coefficient as a distance metric with the Support Tree method of bootstrapping implemented in TMEV used to test the reliability of the clustering patterns (500 bootstrap re-samplings). A second method for clustering strains was developed based on calculating pair-wise genetic similarities in gene conservation profiles by using trinary thresholding of LR data [31], with a score of 1 given to all gene conservation matches (i.e. conserved, divergent of absent in both strains), a score of 0.5 given to absent/divergent pairs, and a score of 0 given to all other mismatches. The genetic similarity was then calculated by dividing the total score of all genes in the array by the total possible score. A custom VBA script for MS-Excel was written to calculate all pair-wise genetic distances (i.e. genetic distance = 1 – genetic similarity) and to calculate bootstrapped distance matrices which were used to create a consensus tree using the programs Neighbor and Consense from the phylogenetic inference package Phylip v.3.6 [36]. Phylogenetic trees were visualized using Treeview v.1.6.6 [37].

### MLST analysis

MLST was performed using the methods of Dingle et al. [7,22]. PCR amplification of the seven target genes was performed using primers described in the references above. Amplicons were purified with the Qiaquick column purification kit (Qiagen, Mississauga, ON) followed by Autoseq-96 (Molecular Dynamics). Sequencing was performed using the MegaBACE Long Read Matrix (Amersham Biosciences) according to the manufacturer's instructions and reaction products were separated on a MegaBACE 500 sequencer (GE Health Care, Piscataway, NJ). Sequence traces were analyzed using the MegaBACE software. MLST alleles and sequence types (ST) were determined for each strain by querying the *C. jejuni *MLST database at the University of Oxford [38] with the edited sequence data. Dendrograms based on the MLST sequence types were obtained using the method of unweighted pair group with arithmetic mean (UPGMA) implemented in the program START2 version 0.5.10 [39]. BURST analysis [40], as implemented in START2, was used to identify potential clonal complexes composed of strains sharing 5 or more identical alleles. Additional clonal complex assignments were determined by querying the *C. jejuni *MLST database with the allelic profiles of the strains.

### Analysis of gene conservation patterns in newly sequenced *C. jejuni *genomes

Available sequence data from completed (strains NCTC 11168, RM1221, 81–176) and ongoing *C. jejuni *sequencing projects (strains 84–25, HB93-13, 260.94, 269.97, CF93-6) was obtained from NCBI's prokaryotic genome sequencing resource [41] and homology searching of genes in selected loci was performed using the program BLASTP [42]. Visualization of sequence identity levels was performed via heat maps generated using a custom-script written in VBA for MS-Excel.

## Abbreviations

CGH: comparative genomic hybridization; HV: highly variable; HD: highly divergent; MD: moderately divergent; MLST: multi-locus sequence typing; ST: sequence type; CC: clonal complex; SLV: single-locus variant; DLV: double-locus variant; TLV: triple-locus variant; LR: Log Ratio; SLRD: significant Log Ratio difference; LOS: lipo-oligosaccharide biosynthesis locus; FL: flagellar biosynthesis locus; CPS: capsular polysaccharide biosynthesis locus; RM: type I restriction-modification locus.

## Authors' contributions

ENT contributed to study design, designed MCGH experiments, wrote custom Excel VBA scripts, carried out downstream data analysis and visualization, and drafted the manuscript; CCL performed all MCGH experiments and performed preliminary data analysis. JMM performed MLST analysis and assisted with preliminary MCGH analysis. VPJG assisted in data interpretation and drafting of the manuscript. JHEN and KR conceived of the study, and participated in its design and coordination and helped to draft the manuscript. All authors submitted comments on drafts and read and approved the final manuscript.

## Supplementary Material

Additional file 1 Pairwise matrix of SLRDs for the 45 strains in the dataset. The (partial) gene content of strains was assessed by analyzing the CGH data using two sets of empirically determined thresholds [31]: Figure S1: < 1% error rate on "likely conserved" and "likely absent" calls. ; Figure S2: < 1% error rate on "likely conserved" and "likely divergent/absent". Strains have been arranged based on the UPGMA dendogram from analysis of MLST data. Boxes in bold represent pairwise distances between members of the same clonal complex (red branches on dendograms). Click here for file
